# Supplementary material for: Hetero-Porous, High-Surface Area Green Carbon Aerogels for the Next-Generation Energy Storage Applications
Source: Nanomaterials (Basel). 2021 Mar 8;11(3):653. doi: 10.3390/nano11030653 (PMC7999108; doi:10.3390/nano11030653)
Supplement: Supplementary file 1 [file nanomaterials-11-00653-s001.pdf]

Supplementary Materials

# Hetero-Porous, High-Surface Area Green Carbon Aerogels for the Next-Generation Energy Storage Applications

**Bony Thomas [1], Shiyu Geng [1], Mohini Sain [1,2] and Kristiina Oksman [1,2,*]**

[1] Division of Materials Science, Department of Engineering Sciences and Mathematics, Luleå University of Technology, SE-971 87 Luleå, Sweden; bony.thomas@ltu.se (B.T.); shiyu.geng@ltu.se (S.G.); m.sain@utoronto.ca (M.S.)
[2] Mechanical & Industrial Engineering (MIE), University of Toronto, Toronto, ON M5S 3G8, Canada
* Correspondence: kristiina.oksman@ltu.se; Tel.: +46-(0)920-493371

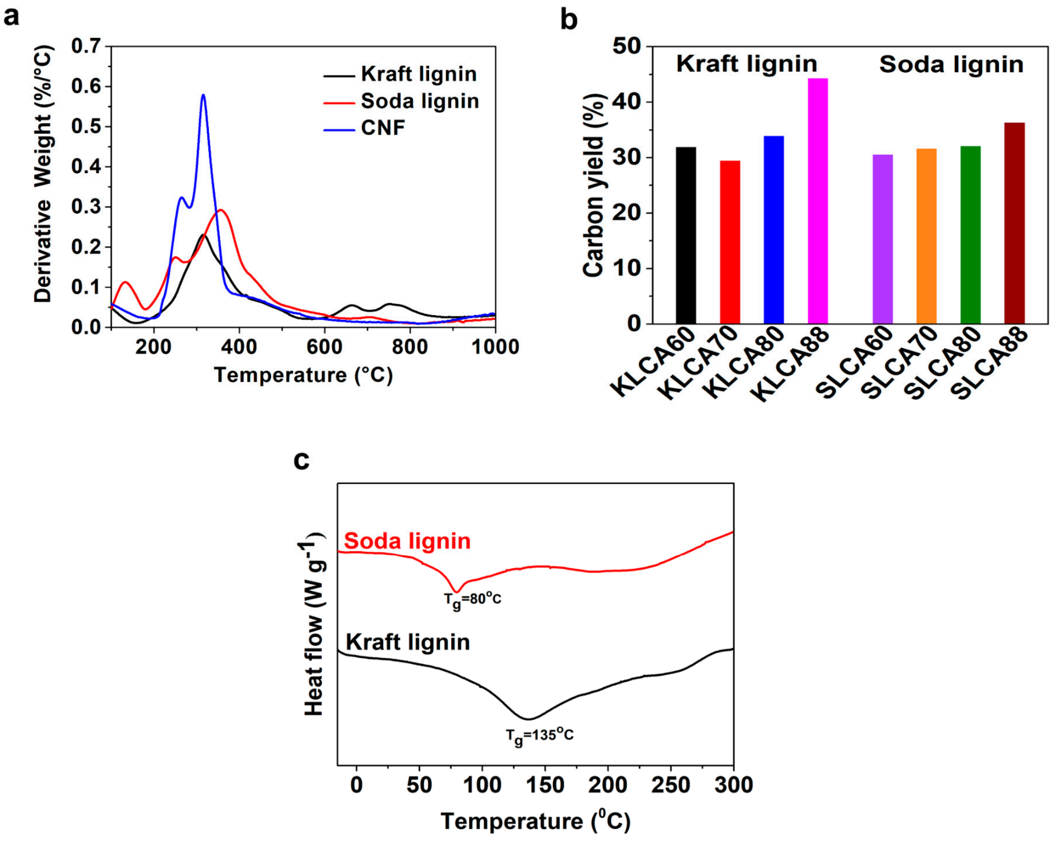


**Figure S1.** (a) Derivative thermogravimetric (DTG) curve of kraft, soda and CNF; (b) carbon yield for KL- and SL-based carbon aerogels; (c) differential scanning calorimetry (DSC) curves for kraft lignin and soda lignin.

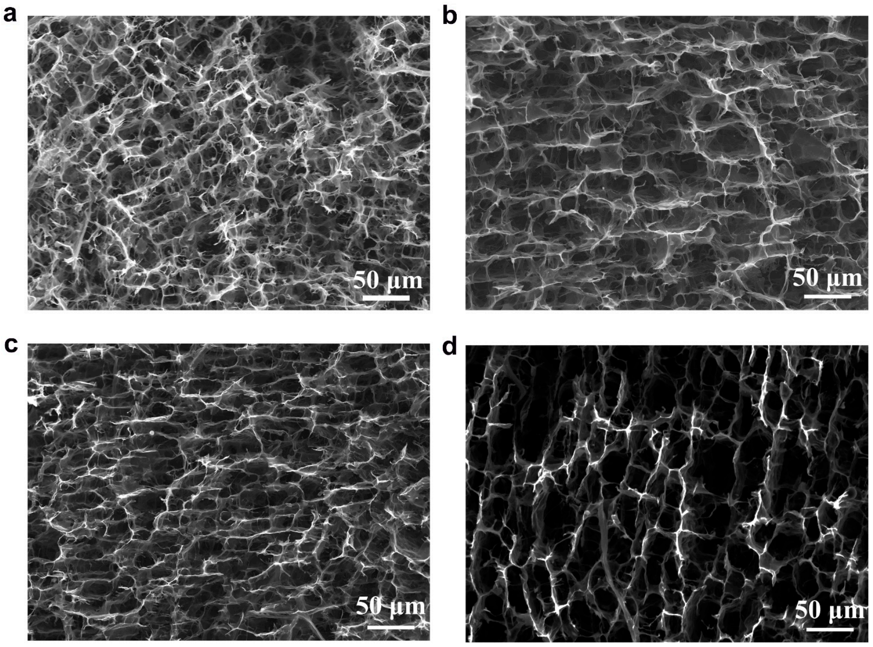


**Figure S2.** Honeycomb-like micro-structure in the cross section of CAs (a) SLCA60 (b) SLCA70 (c) SLCA80 and (d) SLCA88.

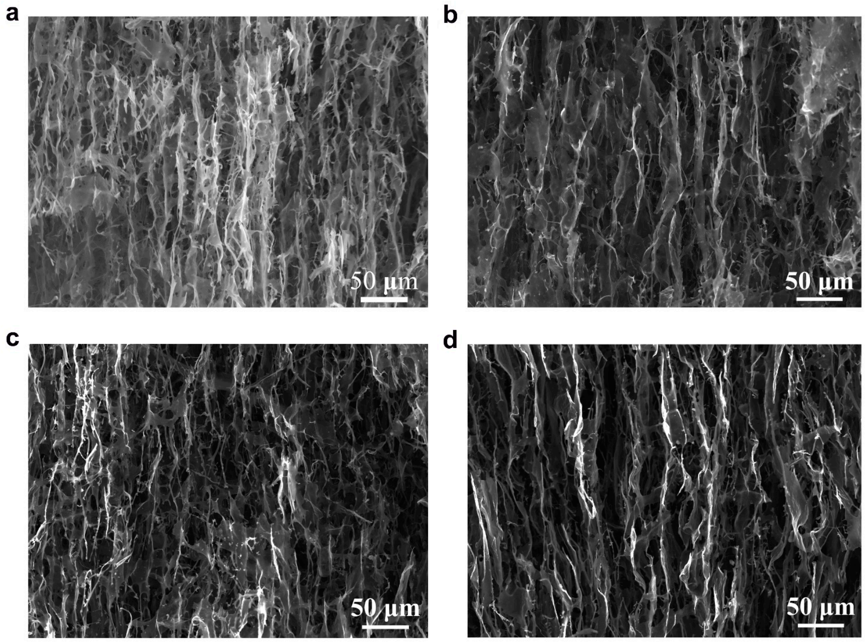


**Figure S3.** Longitudinal channel like micro-structure in the CAs (a) SLCA60 (b) SLCA70 (c) SLCA80 and (d) SLCA88.

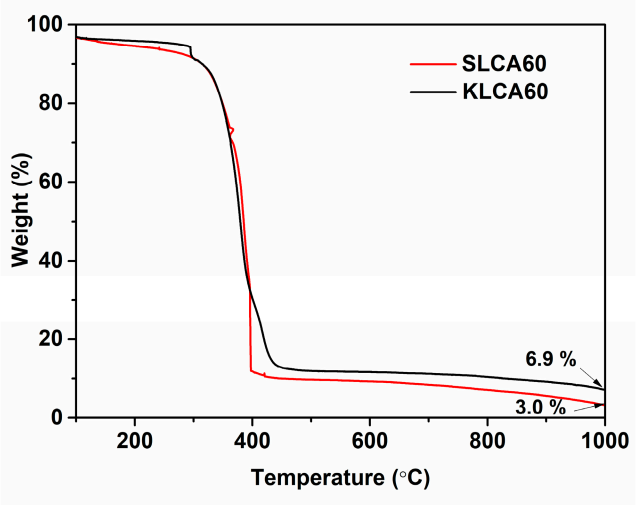


**Figure S4.** Thermogravimetric analysis of SLCA60 and KLCA60 in oxygen atmosphere.

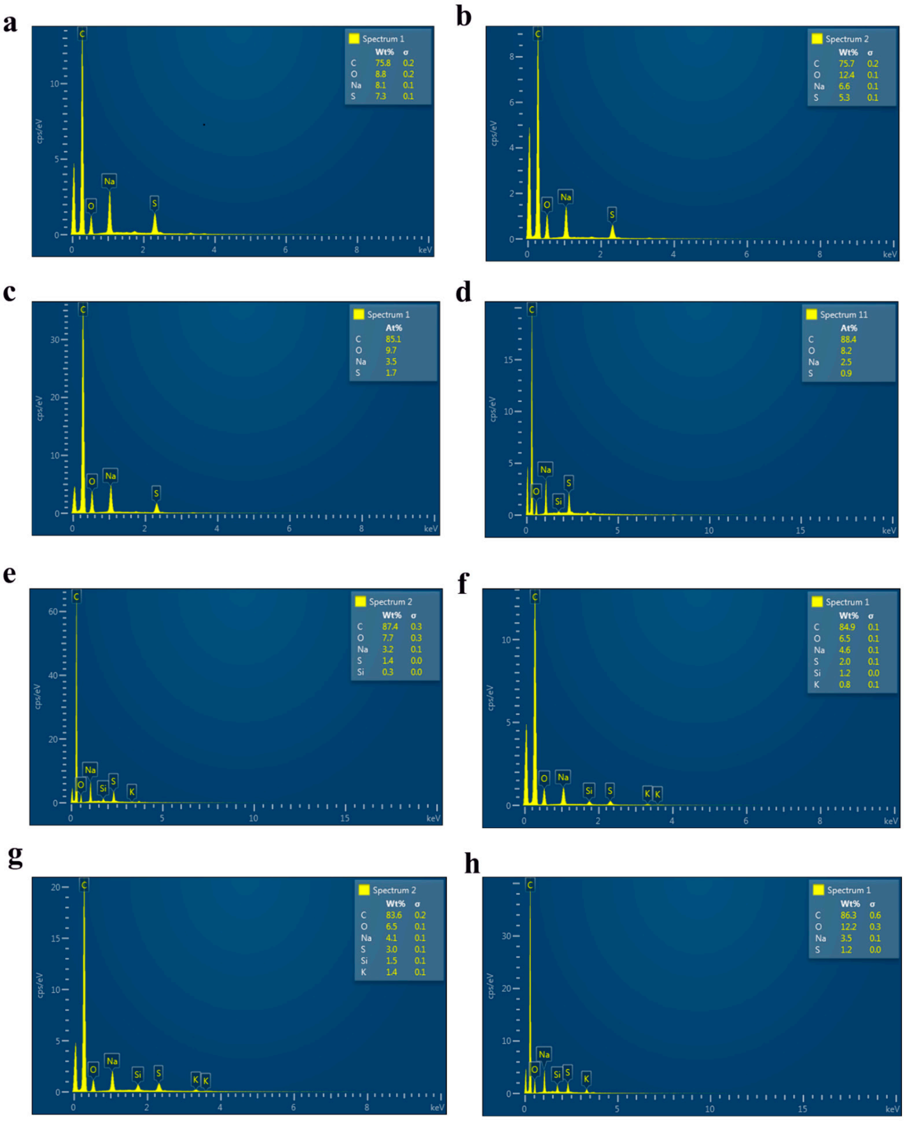


**Figure S5.** EDX spectra for (a) KLCA60, (b) KLCA70, (c) KLCA80 (d) KLCA88 (e)SLCA60 (f) SLCA70, (g)SLCA80, and (h) SLCA88.

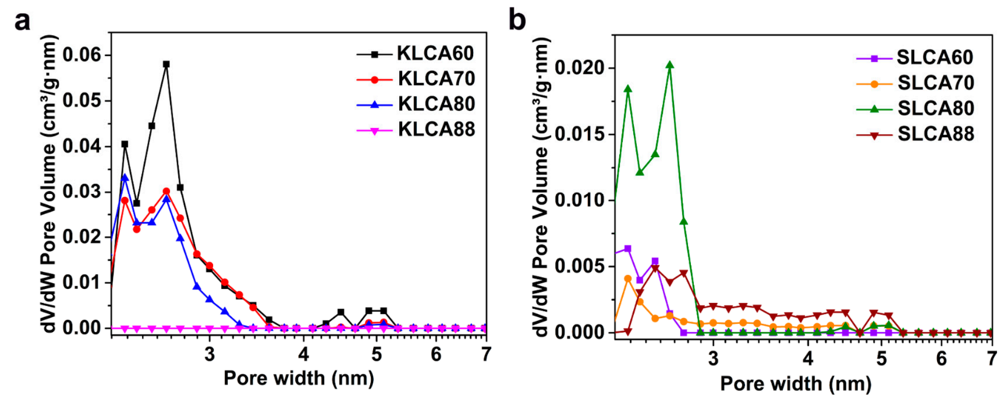


**Figure S6.** Pore size distribution of a) KLCAs and b) SLCAs (represented as differential pore volume plotted against pore width) derived from adsorption isotherms of KLCAs and SLCAs and calculated with the NLDFT model.

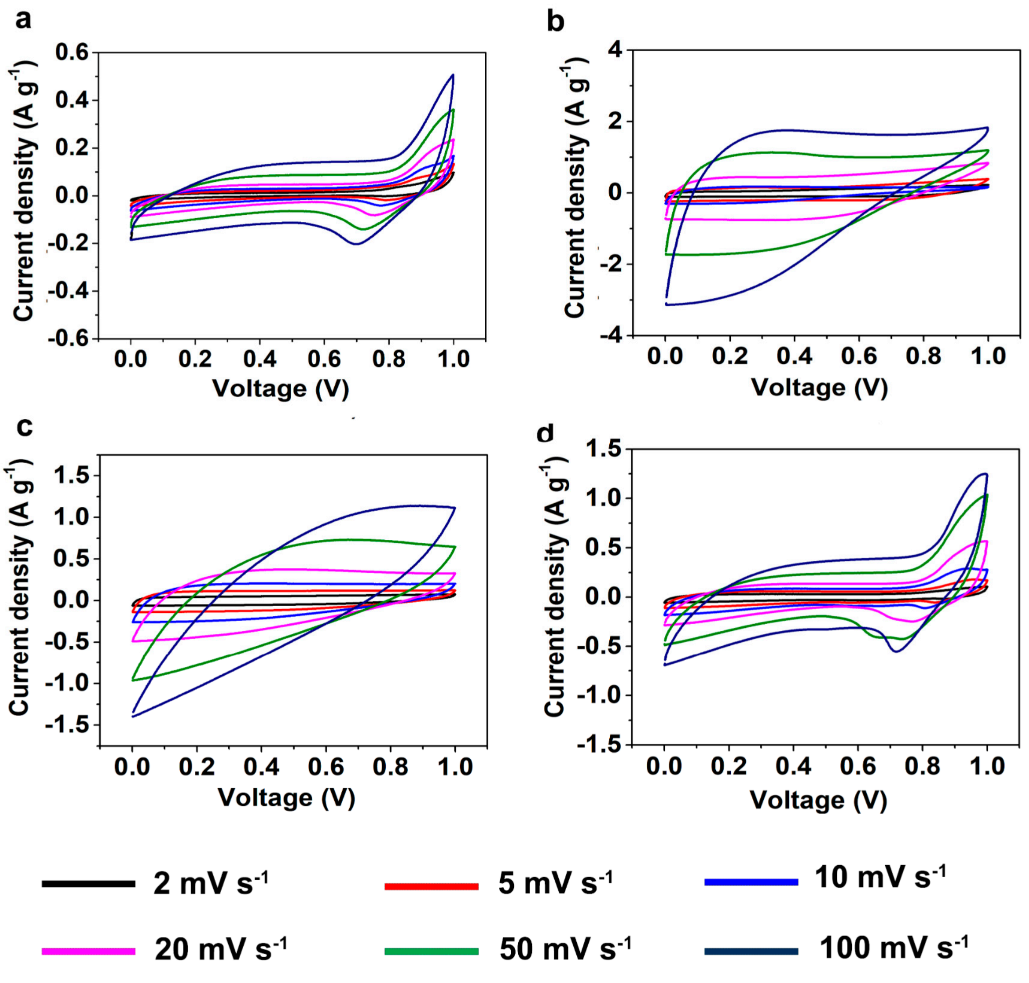


**Figure S7.** Cyclic voltammograms (CVs) and galvanostatic charge discharge (GCD) curves for soda lignin based carbon aerogels. (a) SLCA60 (b) SLCA70 (c) SLCA80 and (d) SLCA88.

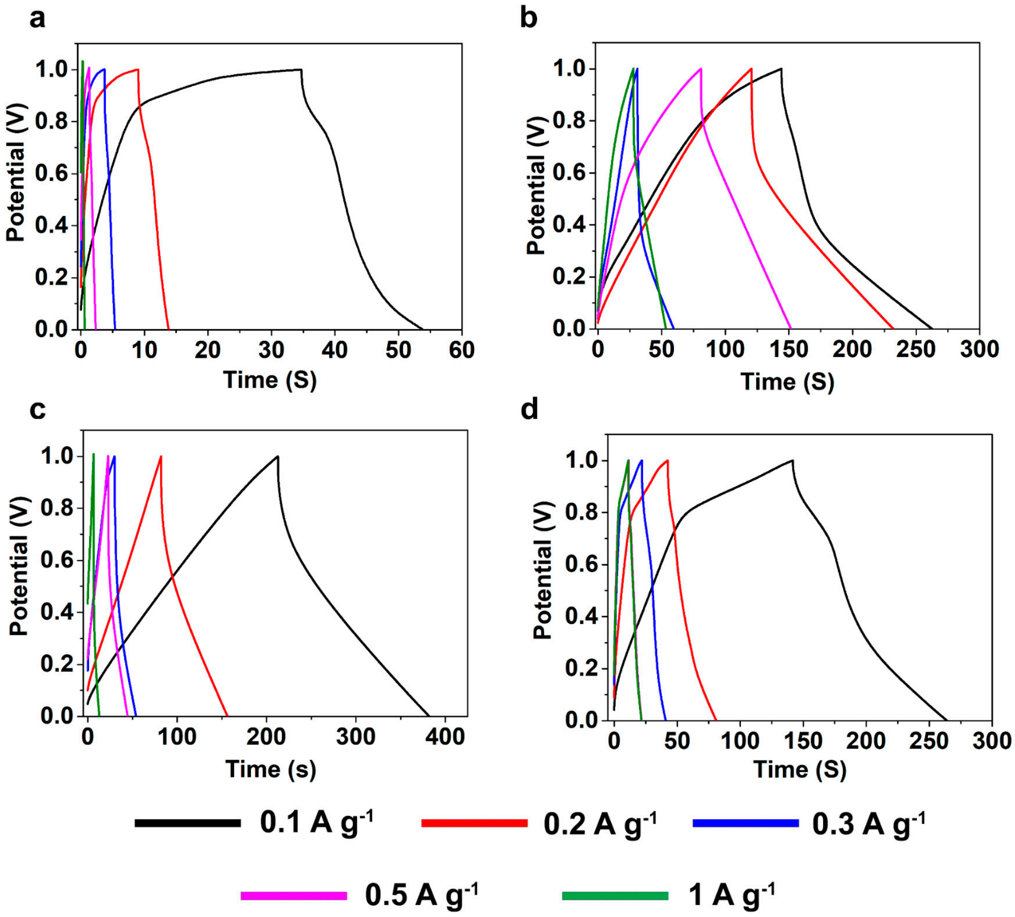


**Figure S8.** Galvanostatic charge discharge (GCD) curves for soda lignin based carbon aerogels. (a) SLCA60 (b) SLCA70 (c) SLCA80 and (d) SLCA88.

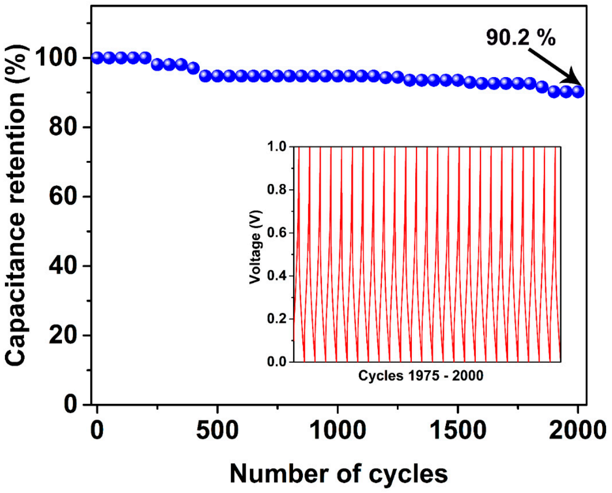


**Figure S9.** Cyclic stability of KLCA60 electrodes after 2000 charge-discharge cycles.

**Table S1.** Electrochemical properties of the supercapacitors of KLCA electrodes.

| | Galvano static charge discharge measurements | | | | Cyclic voltammetry | | |
|---|---|---|---|---|---|---|---|
| **Sample** | **Current density A g$^{-1}$** | **Specific capacitance F g$^{-1}$** | **Energy density Wh kg$^{-1}$** | **Power density W kg$^{-1}$** | **Scan rate mV s$^{-1}$** | **Specific capacitance F g$^{-1}$** | **Energy density Wh kg$^{-1}$** |
| KLCA60 | 0.1 | 162 | 5.7 | 50 | 2 | 163 | 5.7 |
| | 0.2 | 128 | 4.4 | 100 | 5 | 140 | 4.4 |
| | 0.3 | 115 | 4.0 | 150 | 10 | 123 | 4.0 |
| | 0.5 | 111 | 3.8 | 250 | 20 | 108 | 3.8 |
| | 1 | 97 | 3.4 | 500 | 50 | 86 | 3.4 |
| KLCA70 | 0.1 | 105 | 3.7 | 50 | 2 | 129 | 4.5 |
| | 0.2 | 94 | 3.3 | 100 | 5 | 102 | 3.5 |
| | 0.3 | 77 | 2.7 | 150 | 10 | 93 | 3.2 |
| | 0.5 | 66 | 2.3 | 250 | 20 | 71 | 2.5 |
| | 1 | 34 | 1.2 | 500 | 50 | 34 | 1.2 |
| KLCA80 | 0.1 | 79 | 2.7 | 50 | 2 | 95 | 3.3 |
| | 0.2 | 71 | 2.5 | 100 | 5 | 85 | 3.0 |
| | 0.3 | 69 | 2.4 | 150 | 10 | 76 | 2.6 |
| | 0.5 | 62 | 2.2 | 250 | 20 | 63 | 2.2 |
| | 1 | 44 | 1.5 | 500 | 50 | 39 | 1.4 |
| KLCA88 | 0.1 | 93 | 3.2 | 50 | 2 | 106 | 3.7 |
| | 0.2 | 76 | 2.6 | 100 | 5 | 93 | 3.2 |
| | 0.3 | 66 | 2.3 | 150 | 10 | 81 | 2.8 |
| | 0.5 | 49 | 1.7 | 250 | 20 | 67 | 2.3 |
| | 1 | 47 | 1.6 | 500 | 50 | 48 | 1.7 |
